# Identifying predictors of on-table adaptation for pancreas stereotactic body radiotherapy (SBRT)

**DOI:** 10.1016/j.ctro.2023.100603

**Published:** 2023-02-22

**Authors:** Trudy C. Wu, Stephanie M. Yoon, Minsong Cao, Ann C. Raldow, Michael Xiang

**Affiliations:** Department of Radiation Oncology, University of California Los Angeles, Los Angeles, CA, USA

**Keywords:** Pancreatic cancer, Stereotactic body radiation therapy, Adaptive planning, MRI-guided radiotherapy

## Abstract

**Purpose:**

To identify any clinical or dosimetric parameters that predict which individuals may benefit from on-table adaptation during pancreas stereotactic body radiotherapy (SBRT) with MRI-guided radiotherapy.

**Methods and materials:**

This was a retrospective study of patients undergoing MRI-guided SBRT from 2016 to 2022. Pre-treatment clinical variables and dosimetric parameters on the patient’s simulation scan were recorded for each SBRT course, and their ability to predict for on-table adaptation was analyzed using ordinal logistic regression. The outcome measure was number of fractions adapted.

**Results:**

Sixty-three SBRT courses consisting of 315 fractions were analyzed. Median prescription dose was 40 Gy in five fractions (range, 33–50 Gy); 52% and 48% of courses were prescribed ≤40 Gy and >40 Gy, respectively. The median minimum dose delivered to 95% (D95) of the gross tumor volume (GTV) and planning target volume (PTV) was 40.1 Gy and 37.0 Gy, respectively. Median number of fractions adapted per course was three, with 58% (183 out of 315) total fractions adapted. On univariable analysis, the prescription dose (>40 Gy vs ≤40 Gy), GTV volume, stomach V20 and V25, duodenum V20 and dose maximum, large bowel V33 and V35, GTV dose minimum, PTV dose minimum, and gradient index were significant determinants for adaptation (all p < 0.05). On multivariable analysis, only the prescription dose was significant (adjusted odds ratio 19.7, p = 0.005), but did not remain significant after multiple test correction (p = 0.08).

**Conclusions:**

The likelihood of needing on-table adaptation could not be reliably predicted a priori using pre-treatment clinical characteristics, dosimetry to nearby organs at risk, or other dosimetric parameters based on the patient’s anatomy at the time of simulation, suggesting the critical importance of day-to-day variations in anatomy and increasing access to adaptive technology for pancreas SBRT. A higher (ablative) prescription dose was associated with increased use of adaptation.

## Highlights

• A prescription dose >40 Gy in MRI-guided pancreas stereotactic body radiation therapy was associated with increased use of on-table adaptation.
• Clinical variables such as BMI, tumor location, technical resectability status and presence of vessel involvement are not reliable predictors of on-table adaptation.
• Dosimetric variables such as the gross tumor volume (GTV) size, planning target volume (PTV) size, minimum dose delivered to 95% (D95) of the GTV and PTV, GTV minimum, PTV minimum, conformity index, heterogeneity index, gradient index, and dosimetric parameters to organs at risk (OARs) (the absolute volume of an OAR receiving 20–35 Gy and dose maximum) are not reliable predictors of on-table adaptation.
• These findings emphasize the influential magnitude of stochastic day-to-day variations in patient anatomy, which can trigger on-table adaptation for a majority of fractions.
• On-table adaptive technology should be considered when delivering ablative doses to the pancreas.

## Introduction

A minority of patients with pancreatic cancer are diagnosed with localized disease and offered surgery, which provides the highest chance of durable disease control. Despite surgical resection, five-year overall survival remains grim at 10% [1]. Furthermore, tumor resectability is graded on a spectrum, and about one in five patients has true resectable disease at diagnosis [2]. Evaluating candidacy for a margin negative resection (R0) can be challenging and usually half of patients predicted to have R0 resection are found to have microscopic positive margins (R1) [3], [4]. Over recent years, there has been a paradigm shift toward a neoadjuvant approach with systemic therapy +/- radiation in an effort to downstage, improve surgical resectability, and treat occult micrometastatic disease upfront.

Radiation can be employed in the neoadjuvant, definitive, or adjuvant setting. In the neoadjuvant or definitive settings, hypofractionation with stereotactic body radiation therapy (SBRT) has resulted in similar or improved disease outcomes compared to conventionally fractionated radiation while the shorter treatment courses are more logistically convenient and integrate better with systemic therapy [5], [6]. Modern SBRT techniques facilitate the delivery of high doses of radiation to the target tumor, with narrow margins and a steep dose falloff to spare nearby organs at risk (OARs). However, in the upper abdomen, concerns over intra- and inter-fraction motion of the tumor and neighboring gastrointestinal (GI) mucosal structures remain a challenge, and the proximity of the target to sensitive normal tissues has constrained the radiation doses able to be safely delivered [7]. Also, in light of recent results from the phase II trial Alliance A021501, which randomly assigned patients with borderline resectable disease to 8 cycles of modified FOLFIRINOX alone versus 7 cycles of mFOLFIRINOX plus pancreas SBRT, the role of radiation – at least in this clinical scenario – continues to be debated [8], [9], [10], [11].

MRI-guided radiotherapy (MRgRT) may help to overcome many of the challenges of pancreas SBRT by offering real-time, on-board imaging to facilitate precise radiation delivery to soft tissue targets, while minimizing dose to nearby OARs. With the added benefit of online adaptive replanning to account for daily anatomical variations of sensitive OARs, stereotactic magnetic resonance-guided adaptive radiotherapy (SMART) has the potential of enabling higher, ablative-level radiation doses to be safely delivered to pancreatic tumors with increased precision and improved therapeutic ratio. This approach is being studied prospectively in an ongoing phase II trial [12].

Currently, there is limited availability of MR-guided radiotherapy units, and most centers are not equipped to offer on-table adaptive replanning. Even at institutions that can employ SMART, there remains uncertainty over how many fractions (if any) will require online adaptation in any given patient. If providers were able to reliably identify in advance which patients may require one or more fractions adapted, a referral to or treatment at a center with SMART expertise may be anticipated, especially as there is growing interest in dose escalation of pancreas radiation, allowing more optimal use of limited resources. In this study, we asked whether any pre-treatment clinical variables or dosimetric parameters can predict a patient’s need for on-table adaptation while undergoing MRI-guided pancreas SBRT.

## Methods and materials

### Cohort identification

Patients were identified via retrospective chart review at this institution. All patients were treated using MRI-guided adaptive SBRT to a dose of 33–50 Gy in 5 fractions (92% of patients received 40–50 Gy), delivered on non-consecutive days, to intact pancreas between the years of 2016 and 2022. All patients had primary pancreas adenocarcinoma. This study was approved by the institutional review board (IRB).

### Treatment procedure

Targets were gross disease with a planning target volume (PTV) margin selected at the discretion of the treating physician (generally 3 mm); elective nodal radiation was not performed. Patients were simulated and treated in deep inhale breath hold, with the use of respiratory gating, and were instructed to be NPO for 3 h prior to simulation and treatments. Goal PTV coverage during planning was 95%, but this was decreased as needed to meet normal tissue constraints. Planning dosimetric objectives to normal tissues focused on a V33-35 Gy < 0.5–1.0 cc to mucosal GI OARs (duodenum, small bowel, stomach, large bowel), with the specific constraints used depending on the treating physician; liver volume receiving <15 Gy to be >1000 cc, and each kidney V14 Gy < 33%. The ViewRay MRIdian cobalt system (ViewRay Inc. Oakwood Village, OH) was used from 2016 to 2019, and the MRIdian linear accelerator (LINAC) was used from 2019 to 2022.

Standard workflow for adaptive MRgRT is to evaluate dose to GI OARs based on the “anatomy of the day” and adapt if, and only if, the prespecified GI OAR constraints are exceeded. For treatments, a setup MRI scan was acquired, and this was reviewed by the “doctor of the day” (an attending physician designated with checking all SBRT setups), medical physicist, and dosimetrist. The decision to recontour GI OARs on the setup MRI was ultimately at the discretion of the supervising physician; for example, if normal tissues were in a different or closer position to the target compared to the original planning MRI, then recontouring was performed, whereas if normal tissues moved farther away from the target, then the physician may have deemed recontouring (and adaptation) to be unnecessary. Then, the dose to the recontoured OARs was predicted from the original (scheduled) plan, and adaptation was triggered if, and only if, the prespecified GI OAR constraints (in all cases, identical to those originally used for planning) were exceeded based on the “anatomy of the day”. Finally, the dosimetry of the original and adapted plans was compared prior to treatment delivery.

### Study variables

Clinical variables were patient age, sex, body mass index (BMI), tumor location (head, body/tail), technical resectability status per multi-disciplinary tumor board or as defined by consensus guidelines [13], and presence of any vessel involvement (as detected on the most recent diagnostic computed tomography [CT] scan prior to the simulation scan). Dosimetric variables were prescription dose, gross tumor volume (GTV) size, PTV size, minimum dose delivered to 95% (D95) of the GTV and PTV, GTV dose minimum, PTV dose minimum, conformity index, heterogeneity index, gradient index, and additional dosimetric parameters to GI OARs based on the patient’s anatomy at the time of simulation. The GI OARs were stomach, duodenum, small bowel (non-duodenum), and large bowel. Dosimetric parameters were the absolute volume of the organ receiving 20–35 Gy (i.e., V20, V25, V30, V33, V35), and the dose maximum received by the organ, defined as the D0.03 cc of the contour. Conformity and heterogeneity indices were analyzed as percentage points over 1 in the regression models.

#### Statistical analysis

To identify predictors for the number of adapted fractions, ordinal logistic regression was performed, using ordered categories corresponding to the number (0–5) of fractions adapted in the patient’s treatment course. This approach was chosen because it incorporates information regarding the number of fractions adapted per patient, whereas a binomial regression loses that information (all patients with at least 1 fraction adapted are treated as a single group). All study variables were first evaluated in univariable analysis. Then, variables with p-value < 0.10 were selected for inclusion in a multivariable model. Finally, Bonferroni multiple test correction was performed on the results in the multivariable model. Results were ultimately considered significant at p < 0.05 in the multivariable model after Bonferroni correction. Analyses were performed using MATLAB version R2021a.

## Results

A total of 63 patients were identified, representing 63 treatment courses. Twenty-six (41.3%) patients were male, and 37 patients (58.7%) were female with a median BMI of 23.2 (IQR 21.5–26.3; range, 16.2–39.0). Tumor targets were located in the head and body/tail of the pancreas in 38 (60.3%) and 25 (39.7%) of patients, respectively. The median prescription dose was 40 Gy in five fractions (range, 33–50 Gy); 52.4% of courses were prescribed ≤40 Gy, and 47.6% of courses were prescribed >40 Gy. Table 1 lists the detailed characteristics of the patients and treatment courses. For the main dosimetric characteristics, the median GTV D95 was 40.1 Gy (IQR 36.2–42.7; range, 25.3–57.9), GTV dose minimum 30.0 Gy (IQR 26.0–36.2; range, 18.5–52.3), PTV D95 37.0 Gy (IQR 34.2–40.7; range, 23.3–51.6), and PTV dose minimum 27.0 Gy (IQR 23.2–31.1; range, 13.3–42.9). Table 2 lists the detailed dosimetric characteristics.

**Table 1:** Patient characteristics of entire cohort (n = 63).

	Patients (%)
Total Prescribed Dose	
≤ 40 Gy	33 (52.4)
> 40 Gy	30 (47.6)
Dose per Fraction	
6.6 Gy	4 (6.35)
7 Gy	1 (1.59)
8 Gy	28 (44.4)
10 Gy	30 (47.6)
Age (median, range)	68 (39–83)
Gender	
Male	26 (41.3)
Female	37 (58.7)
BMI (median, range)	23.2 (15.2–39.0)
Tumor Location	
Head	38 (60.3)
Body/tail	25 (39.7)
Resectability Status	
Resectable	8 (12.7)
Borderline	14 (22.2)
Unresectable	41 (65.1)
Vessel Involvement	
Yes	54 (85.7)
No	9 (14.3)
GTV volume (cc) (median, IQR)	38.0 (21.7–56.3)
PTV volume (cc) (median, IQR)	69.1 (42.0–85.4)

Gy, gray; BMI, body mass index; IQR, interquartile range; GTV, gross tumor volume; PTV, planning target volume; cc, cubic centimeters.

**Table 2:** Dosimetric characteristics of the 63 treatment courses.

	Median	IQR	Range
Stomach V20 (cc)	15.72	5.24–27.81	0.00–111.24
Stomach V25 (cc)	4.60	0.90–7.97	0.00–37.88
Stomach V30 (cc)	0.68	0.04–1.48	0.00–11.60
Stomach V33 (cc)	0.11	0.00–0.24	0.00–5.63
Stomach V35 (cc)	0.01	0.00–0.09	0.00–3.35
Stomach Dmax (Gy)	34.18	30.34–36.18	2.47–42.87
Duodenum V20 (cc)	17.11	6.60–26.65	0.00–89.39
Duodenum V25 (cc)	5.51	2.08–11.00	0.00–55.67
Duodenum V30 (cc)	0.79	0.33–2.87	0.00–23.55
Duodenum V33 (cc)	0.15	0.00–0.89	0.00–7.74
Duodenum V35 (cc)	0.02	0.00–0.29	0.00–5.01
Duodenum Dmax (Gy)	34.81	32.34–37.57	12.25–50.57
Small bowel V20 (cc)	6.10	2.43–20.83	0.00–162.15
Small bowel V25 (cc)	2.29	0.39–5.24	0.00–78.25
Small bowel V30 (cc)	0.21	0.00–1.13	0.00–37.21
Small bowel V33 (cc)	0.02	0.00–0.28	0.00–21.67
Small bowel V35 (cc)	0.00	0.00–0.09	0.00–13.40
Small bowel Dmax (Gy)	32.88	27.64–36.43	2.63–42.78
Large bowel V20 (cc)	8.20	1.51–26.34	0.00–92.97
Large bowel V25 (cc)	0.87	0.00–6.48	0.00–34.62
Large bowel V30 (cc)	0.00	0.00–1.01	0.00–6.40
Large bowel V33 (cc)	0.00	0.00–0.21	0.00–2.73
Large bowel V35 (cc)	0.00	0.00–0.05	0.00–1.69
Large bowel Dmax (Gy)	29.08	22.57–35.38	15.27–42.81
GTV D95	40.14	36.17–42.71	25.32–57.92
GTV min	29.95	26.02–36.20	18.51–52.28
PTV D95	36.98	34.20–40.72	23.26–51.58
PTV min	27.03	23.16–31.06	13.30–42.91
Conformity index	1.02	0.80–1.18	0.46–2.26
Heterogeneity index	1.26	1.21–1.28	1.11–1.55
Gradient index	5.28	4.16–6.62	3.31–14.06

IQR, interquartile range; GTV, gross tumor volume; PTV, planning target volume; cc, cubic centimeters.

**Fig. 1:**
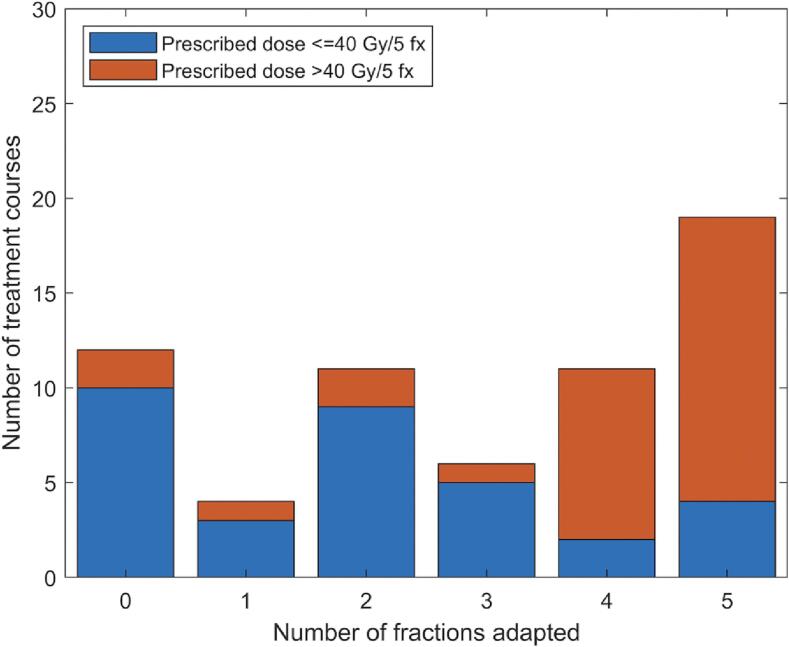
Histogram of the number of fractions adapted for 63 treatment courses of MRI-guided pancreas SBRT. * Due to the association between prescription dose and the use of adaptation, the histogram is color-coded according to the prescription dose.

The median number of fractions adapted was three (IQR 1–5; range, 0–5), with overall 183 of 315 total fractions (58%) adapted. Fig. 1 is a histogram of the number of treatment courses according to the number of fractions adapted within that treatment course, color-coded by the prescribed dose.

Table 3 lists the results of the univariable analysis. Prescribed dose (>40 Gy vs ≤ 40 Gy) was the largest determinant, with odds ratio of 9.79, and the most statistically significant (p < 0.0001). Ten additional variables had p < 0.05: GTV volume, stomach V20 and V25, duodenum V20 and dose maximum, large bowel V33 and V35, GTV dose minimum, PTV dose minimum, and gradient index, and three additional variables had p < 0.10: BMI, PTV volume, and heterogeneity index. Interestingly, small bowel and duodenum V25-V35 Gy parameters were not predictive for the use of adaptation. All 13 variables with a p < 0.10 were further analyzed in the multivariable model (Table 4). Prescribed dose was the only statistically significant determinant, with an adjusted odds ratio of 19.7, and p value of 0.005; however, this did not remain significant after multiple test correction (p = 0.08).

**Table 3:** Univariable analysis of predictors for number of fractions adapted.

	Odds ratio (95% confidence interval)	P-value
Prescribed dose (>40 Gy vs ≤ 40 Gy)	9.79 (3.50–27.38)	<0.0001
Age (per year)	0.98 (0.94–1.03)	0.45
Sex (female vs male)	1.19 (0.49–2.89)	0.70
Body mass index (per 1 unit)	1.09 (0.99–1.21)	0.09
Tumor location (head vs body/tail)	0.55 (0.22–1.36)	0.19
Borderline resectable (vs resectable)	0.52 (0.11–2.46)	0.41
Unresectable (vs resectable)	1.00 (0.26–3.83)	0.99
Vessel involvement	1.32 (0.38–4.61)	0.66
GTV volume	1.02 (1.00–1.04)	0.01
PTV volume	1.01 (1.00–1.02)	0.07
Stomach V20	1.03 (1.01–1.06)	0.02
Stomach V25	1.08 (1.00–1.16)	0.05
Stomach V30	1.02 (0.82–1.28)	0.84
Stomach V33	0.86 (0.50–1.47)	0.58
Stomach V35	0.70 (0.26–1.90)	0.48
Stomach Dmax	0.99 (0.94–1.05)	0.74
Duodenum V20	1.04 (1.01–1.08)	0.02
Duodenum V25	1.02 (0.97–1.08)	0.40
Duodenum V30	0.95 (0.84–1.08)	0.43
Duodenum V33	0.79 (0.58–1.08)	0.14
Duodenum V35	0.62 (0.32–1.18)	0.14
Duodenum Dmax	1.07 (1.01–1.14)	0.03
Small bowel V20	1.02 (0.99–1.04)	0.16
Small bowel V25	1.04 (0.98–1.10)	0.23
Small bowel V30	1.07 (0.94–1.21)	0.29
Small bowel V33	1.14 (0.90–1.45)	0.27
Small bowel V35	1.27 (0.84–1.92)	0.26
Small bowel Dmax	1.02 (0.97–1.08)	0.36
Large bowel V20	1.02 (0.99–1.04)	0.14
Large bowel V25	1.02 (0.96–1.08)	0.49
Large bowel V30	0.90 (0.68–1.19)	0.45
Large bowel V33	0.33 (0.13–0.86)	0.02
Large bowel V35	0.02 (0.00–0.74)	0.03
Large bowel Dmax	1.04 (0.98–1.10)	0.22
GTV D95	0.96 (0.90–1.03)	0.32
GTV min	0.84 (0.78–0.91)	<0.0001
PTV D95	1.01 (0.94–1.09)	0.76
PTV min	0.88 (0.82–0.96)	0.003
Conformity index	1.00 (0.98–1.01)	0.62
Heterogeneity index	1.07 (0.99–1.15)	0.09
Gradient index	0.71 (0.56–0.89)	0.003

*GTV, gross tumor volume; PTV, planning target volume.

**Odds ratios for volumetric parameters are per 1 increase in cubic centimeters, and odds ratios for dose maximum are per 1 increase in Gy. Odds ratios for conformity index and heterogeneity index are per 1% increase over 1.

**Table 4:** Multivariable analysis of predictors for number of fractions adapted, using the variables from the univariable analysis with p < 0.10.

	Odds ratio (95% confidence interval)	P-value (uncorrected)	P-value (corrected)
Prescribed dose (>40 Gy vs ≤ 40 Gy)	19.68 (2.41–160.99)	0.005	0.08
Body mass index	1.01 (0.89–1.14)	0.92	1
GTV volume	0.97 (0.89–1.05)	0.42	1
PTV volume	1.01 (0.95–1.08)	0.68	1
Stomach V20	1.10 (1.00–1.21)	0.06	0.83
Stomach V25	0.84 (0.66–1.07)	0.16	1
Duodenum V20	1.04 (0.99–1.09)	0.09	1
Duodenum Dmax	1.01 (0.92–1.11)	0.81	1
Large bowel V33	6.50 (0.19–221.05)	0.3	1
Large bowel V35	0.00 (0.00–26.07)	0.16	1
GTV min	0.96 (0.81–1.14)	0.65	1
PTV min	0.87 (0.70–1.07)	0.19	1
Gradient index	1.18 (0.77–1.79)	0.45	1
Heterogeneity index	1.06 (0.97–1.17)	0.2	1

## Discussion

In this study of pancreas SMART without elective nodal coverage, we found only prescription dose to be associated with increased use of on-table adaptation, although this was of borderline statistical significance after multivariable analysis and multiple test correction. This suggests that administering ablative doses is more likely to violate OAR constraints and prompt adaptive re-planning and generation of a newly optimized plan. The other clinical and dosimetric parameters analyzed in this study could not reliably predict individual patient odds for on-table adaptation in advance; thus, emphasizing the critical importance of stochastic day-to-day variations of patient anatomy and organ positions, and the merit of having adaptive planning available to deliver ablative radiation safely and effectively for pancreatic cancer.

Compared to conventionally fractionated radiation, SBRT has several advantages including superior local control, fewer fractions resulting in a markedly shorter treatment time, and minimal interference with systemic therapy [6]. In practice, SBRT is the preferred technique among academic radiation oncologists [14]. Over recent years, there has been growing interest in a total neoadjuvant approach with upfront systemic therapy and radiation prior to surgery. However, the recently published phase 2 trial Alliance A021501 did not find a benefit to the R0 resection rate or overall survival with the addition of pancreas SBRT after mFOLFIRINOX for borderline resectable pancreas cancer [11]. Reasons for this are currently unclear and may be multifactorial, and the role of pancreas SBRT continues to be debated. However, the sub-ablative doses used in the Alliance trial, and the lack of widespread MRgRT with on-table adaptation, may have led to insufficient doses delivered to tumor and higher-than-intended doses delivered to normal tissues, both of which may compromise the therapeutic index.

At our center, all pancreas SBRT (unless the patient is contraindicated to undergo MRI) is delivered on a ViewRay MRIdian linear accelerator with an adaptive process in place determined by prespecified GI OAR constraints [15], [16]. In our study, we determined prescribed dose is the most reliable predictor for on-table adaptation; however, this was not of statistical significance after multiple test correction. In the multivariable analysis, prescription dose >40 Gy resulted in almost a 19-fold increased likelihood of adaptation, as compared to ≤40 Gy, and was selected as the cutoff given expert recommendations to ideally cover the tumor vessel interface to 40 Gy [17]. Additional clinical and dosimetric variables were evaluated, and several dosimetric parameters calculated from the patient’s anatomy at time of simulation scan were significant on univariable analysis (Table 3); however, ultimately, none remained significant in multivariable analysis after multiple test correction (Table 4). Also, even though critical GI OARs were generously spared in most cases during optimization of the original (scheduled) plan, adaptation was still warranted in about 60% of fractions, emphasizing the influential magnitude of stochastic day-to-day variations in patient anatomy. In combining our adaptation rate with similar experiences by Hassanzadeh et al., and Chuong et al., 78.3% (556/710) of SBRT fractions required a reoptimized adaptive treatment plan using SMART [18], [19].

Our findings suggest that pancreas SBRT with greater than 40 Gy in 5 fractions should only be attempted if on-table adaptive planning is available, given three in four patients required adaptation across studies. If the prescribed dose is ≤40 Gy, treatment is less likely to require on-table adaptation. However, it was still not possible to identify, in advance, which of those patients may need adaptation based on key clinical variables or dosimetric parameters from the patient’s simulation scan. In particular, interestingly, small bowel and duodenum V25-V35 Gy were not significantly predictive of the need to adapt. This suggests that, regardless of the prescription dose, patients may generally be best served by receiving treatment at a center with access to on-table adaptive technology, since which patients will need adaptation cannot be reliably identified in advance. Additionally, emerging data suggest achieving a higher biologic effective dose (BED) can give rise to improved local control [18], [20], [21]. While there is a growing interest in dose escalation for pancreas radiation, safely delivering such doses is challenging as serious early and late GI toxicities have been reported [7], [22], [23], [24]. The intra- and inter-fraction stochastic changes of neighboring OARs, such as the small bowel and stomach, and movement of the tumor can be mitigated with SMART [18]. Overall, our results suggest that adaptive technology with MRgRT should become more widely available, and if it is not available at a particular center, providers may consider referring to a center with such capabilities to deliver safe and effective pancreas SBRT. It should be acknowledged that achieving high BED without SMART but instead, using additional strategies such as breath hold and intrapancreatic fiducials is feasible, but no direct comparisons between the various techniques have been made [25].

A strength of this study is our longstanding institutional experience and familiarity with MRgRT using the ViewRay MRIdian system [26]. Furthermore, our team of physicians, medical physicists, dosimetrists and radiation therapists have a standardized and streamlined adaptive workflow, described here [15], [16]. Our study also has some limitations. Although adaptive criteria were pre-specified, one limitation is the potential for inter-provider variation in clinical judgement and threshold to trigger on-table adaptation among physicians assigned as “doctor of the day”. Furthermore, our center’s adaptive criteria have evolved with time, and were pre-specified at the discretion of the treating physician; thus, also subject to potential inter-provider variations, although they generally were consistent with ensuring V33-35 Gy < 0.5 cc or < 1 cc to mucosal GI OARs. However, we believe that any such variations will be diffused across all the treatments in the entire pooled analysis, as it is unlikely that any provider-specific variations were systematically correlated with baseline clinical or dosimetric parameters. For our study, we also considered analyzing the geometric distance from the GTV/PTV to OARs as a variable of interest; however, a reliable and structured method to determine this distance was not deemed feasible, and we considered dose-volume histogram values of OARs to be an equally informative (if not more so) surrogate to geometric distances. Also, the thinner multileaf collimators and 6 MV flat filtering free beam on the MRIdian LINAC may confer in better dose conformity and less low-dose spill compared to the cobalt system; however, it is unlikely that variations between the two delivery systems greatly impacted the final and approved radiation plan. Finally, as this was a single-institution study, it is worth replicating our study across additional institutions to ensure generalizability of the results.

## Conclusions

Utilizing SMART at our center, we determined ablative pancreas SBRT doses were associated with increased likelihood to warrant on-table adaptation. Additional key clinical variables and dosimetric parameters were not predictive. With growing interest in dose escalation to improve local control and margin status, the utilization of adaptive technology may become increasingly important in pancreas SBRT.

## Declaration of Competing Interest

The authors declare the following financial interests/personal relationships which may be considered as potential competing interests: Ann C. Raldow reports a relationship with Intelligent Automation Inc that includes: consulting or advisory. Ann C. Raldow reports a relationship with ViewRay Inc that includes: consulting or advisory. Ann C. Raldow reports a relationship with Varian Medical Systems Inc that includes: speaking and lecture fees. Ann C. Raldow reports a relationship with Clarity PSO/RO-ILS RO-HAC that includes: speaking and lecture fees. Ann C. Raldow reports a relationship with ViewRay Inc that includes: funding grants. Ann C. Raldow reports a relationship with Veteran’s Health Administration Radiation Oncology Quality Surveillance Program Services that includes: panel membership. Minsong Cao reports a relationship with ViewRay Inc that includes: consulting or advisory. Trudy C. Wu reports a relationship with ViewRay Inc that includes: paid expert testimony.
